# Institutional similarity drives cultural similarity among online communities

**DOI:** 10.1038/s41598-022-23223-8

**Published:** 2022-11-08

**Authors:** Qiankun Zhong, Seth Frey

**Affiliations:** Department of Communication, University of California, Davis, USA

**Keywords:** Social evolution, Computational science

## Abstract

Human organizations are driven by their rules and cultures. But the effects of rules and cultures on organizational development cannot be understood without untangling their effects on each other. People’s values are contingent on how they have been enculturated within organizations. Conversely, their values may influence the organizations they join, particularly in online community settings, where users have thousands of organizations to choose from and exert selection pressure in favor of communities with favorable rules. Using longitudinal data on the rules systems of thousands of online communities, as well as the traffic of millions of users between them, we use techniques from network science to disentangle the relationship between cultural assimilation and institutional assimilation. We find that institutional similarities in administrative rules and informational rules drive cultural similarities. We discuss implications of these findings for research on organizational evolution, institution and culture, and the use of tracking data in organizational studies.

## Introduction

How communities and organizations develop depends greatly on their institutions and cultures. Culture provides community members with a group identity and behavioral guidance, while institutions constitute a set of structuring rules that constrain members’ behaviors. Yet, it is difficult to disentangle the effects of institutions and cultures on a community’s evolution due to their mutual effects on each other. For example, Alesina, Cozzi, and Mantovan show that various preindustrial institutions can lead to long-lasting cultural differences in people’s perceptions of poverty and wealth^1^. These differences in cultural values in turn influence policy choices and their effects today. Thus, to understand how institutions and cultures affect community development over time, we have to investigate the relationship between these two factors. Indeed, the importance of the relationship between institutions and culture has been widely recognized in economics^2–4^, sociology^5–8^, anthropology^9–11^, political science^12^ and communication^13–15^.

Cultural effects are typically more difficult to detect than institutional effects because “top-down” institutional changes work more directly and are easier to operationalize and observe than changes in culture. Individuals in most institutional or organizational settings do not have any power over those institutions, making it more difficult for their cultural preferences to directly influence institutional development. However, there is one domain in which the tangled relationship between culture and institutions is more direct, observable, and mutual: online communities. Online communities are an ideal laboratory for understanding the mutual effects of institutions and culture for three reasons: (1) they are similar to real-world communities in that institutions directly regulate community members’ behaviors, and members can internalize the rules into their cultural values and preferences^4,16–18^; (2) they are different from real-world communities in that members of online worlds can choose to migrate between communities at low cost, offering them more bargaining power over institutional structure. Community members can then select against or directly shape the institution’s development towards their own cultural values and preferences. (3) Users who self-select into the same communities share a sense of identity and preferences that form the community group culture, making it possible to infer similar cultural preferences from communities that share overlapping group membership. This observable pattern provides a unique lens into the effects of culture on a community’s formal rules.

### Player traffic between customized self-governing Minecraft servers

The video game Minecraft provides a useful context for discussing the relationship between subcommunities and their rules. Minecraft is a massive multiplayer online game that allows for various autonomous user activities, including building with blocks, exploring a virtual world, gathering resources, exchanging goods, and engaging in game combat. Importantly, in Minecraft, users can establish and manage their own private servers for playing the game. These servers function both as spaces other users can explore and communities that users can engage with^19^. Someone who sets up a server, the system administrator, takes the responsibility of governing it. To achieve success at building a community of players around their server, administrators have to recruit and retain repeat visitors who can and do migrate between servers at a low cost. Administrators also face constraints in physical resources (e.g., RAM, CPU, bandwidth, monthly server fees) and virtual resources (e.g., software-based currency, reputation systems), all of which must be carefully managed to provide the membership with a quality game experience. Minecraft has been used in science for education^20,21^, design^22,23^, and the study of self-governance^19,24^.

### Rules in Minecraft

In the Minecraft ecosystem, administrators who run private servers rely on custom software-based institutions to manage limited resources and solve collective action problems. These software “plugins” are modular programs that administrators can install on their servers to automatically implement rules and other political-economic constructs. Plugins can allow for certain behaviors or activities, or improve the experience of them. For example, “factions” is a plugin that allows administrators to socially subdivide their community. Others prohibit or punish rule violations. For example, the “AntiCheat” plugin prohibits cheating behavior in the game, while “Combatlog” is used topunish unwelcome aggression. By “mixing and matching” plugins and fine-tuning their settings, server administrators craft highly customized formal institutions and implement a social structure that can solve problems and achieve governing goals.

As of 2016, when our data collection ended, the Minecraft community has developed almost 20,000 plugins categorized under 16 types by the Minecraft developer community. (The plugin categories are Admin Tools, Anti-griefing Tools, Chat Related, Developer Tools, Economy, Fixes, Fun, General, Informational, Mechanics, Role Playing, Teleportation, Website Administration, World Editing and Management, World Generators, and Miscellaneous. See https://www.curseforge.com/minecraft/bukkit-plugins/world-editing-and-management.) Among those, Frey and Sumner^19^ identified four types that directly related to governance: top-down administration, communication, economy, and information.

Plugins in the administration category allow administrators to execute additional control over server states and player behavior toward preventing or remediating problem behaviors among the games anonymous and young users. Plugins like WorldGuard permit the administrator to manage vandalism by rolling parts of the world back to prior snapshots, while GroupManager and Nations help administrators distribute administrative burdens over a hierarchy of "moderator" users with elevated rights. Plugins in the communication category facilitate interpersonal communication by providing additional or higher bandwidth channels for peer-to-peer communication. For example, the popular “Dynmap” plugin renders a dynamic web-based map of the entire world that players use to coordinate their actions and find each other. Informational plugins provide more channels for broadcasting messages and regulations to the community. For example, the “AutoMessage” plugin makes it easier for administrators to send specific contextual information to users automatically in response to environmental triggers, while “LogBlock” helps players resolve conflicts on their own by encoding by making publicly accessible all prior changes to all locations of the world. Economy plugins protect private property rights and facilitate resource exchange. Plugins like “iConomy”, “ChestShop”, and “Signshop” all support market exchange, either peer-to-peer or peer-to-administrator, while plugins like “Lockette” and “Townies” implement private property rights on top of Minecraft's common property default.

### Culture and membership in Minecraft

Drawing from one element of the sociological conceptualization of culture, we understand the culture in Minecraft as a set of cultural repertoires based on shared practices and experiences, and isolate it behaviorally in terms of the server communities that players self-select into: a major part of the ecology of the game is that players can choose what community they join. A cultural repertoire is acquired through players’ experiences in different servers and interactions with other players. The communities that a user selects into thus give a sense of the range of identities that the user holds. And when many users share overlap in their range of identities, evidenced by their tendency to traffic in the same subset of servers, we infer that they share this sense of culture, operationalized here both as overlap in the set of subcultures, and frequent interaction over a set of similar communities. To be clear, we are not attempting here to define the cultural identity of users in terms of the communities they visit, nor the subculture of a community by the users who visit it: we are attempting to define a set of communities as culturally similar by the existence of a large and consistent group of users who travel together between them.

Based on our definition of Minecraft culture—repertoires of shared meaning that provide references for preference and behaviors—it is reasonable to measure the cultural similarity between servers by dual membership; servers with a high share of dual membership will share a similar cultural repertoire either because, having advertised on similar markers of identity, they attract the same types of users, or because of the knowledge and practices that their dual-membership users contribute to both group repertoires. Accordingly, servers with a low share of dual membership are less likely to share a similar group repertoire because their members are less likely to have similar identities or experiences in Minecraft.

Given the close connection between shared membership and server culture similarities, two claims become apparent. On one hand, shared membership can lead to institutional similarities. First, shared membership facilitates information transmission in institutional isomorphism, which refers to a process that drives one organization to resemble others in the same environmental condition^5^. DiMaggio and Powell^5^ provide one explanation for the institutional isomorphism in Minecraft, that is, the uncertainty and risk in the organizational environment drive the communities to mimic the institutions of each other. Second, shared membership can influence institutional development through user preferences. Server success is almost solely dependent on recruiting and retaining repeat visitors, so users indeed have the bargaining power to negotiate with administrators about institutional decisions. Through this kind of mechanism, users’ preferences can possibly influence institutional development. Thus,***

*H1: Shared membership between Minecraft servers causes them to become more institutionally similar.*

On the other hand, Institutional similarity can drive shared membership. Individuals’ behavioral and cultural preferences in Minecraft are cultivated and internalized through institutions. When individuals internalize an institutional logic, they may self-select to the institutions close to their previous institutional experience in Minecraft. First, individuals who learned certain behavior in their first Minecraft community might suffer social costs in institutions that do not reward those sets of behaviors. Comparing to learning new behaviors and preferences in a different environment, choosing a similar enough environment provides lower learning cost and higher average payoff. Second, individuals may normalize institutional logic and enforcement, resulting in cultural persistence^18,25^. Although cultural persistence may also happen in Minecraft, given the low cost of migration between Minecraft communities, it is more likely that individuals will migrate to communities that provide similar institutional experiences. Thus,

*H2: Institutional similarity between Minecraft servers causes them to exhibit more shared membership.*

Although they are phrased in a way that may sound mutually exclusive, our method, grounded in network dynamics and dynamical systems, permits an approach under which both or neither may be true simultaneously, as in the case that institutional similarity and shared membership are mutually constitutive.

## Results

### Data

We analyze longitudinal data of user visits and rule changes between week 5 and week 22 in 2016 among 1097 Minecraft servers. Full details of our data processing and analysis are available in the Supplementary text S1.

### Method and analysis

**Figure 1:**
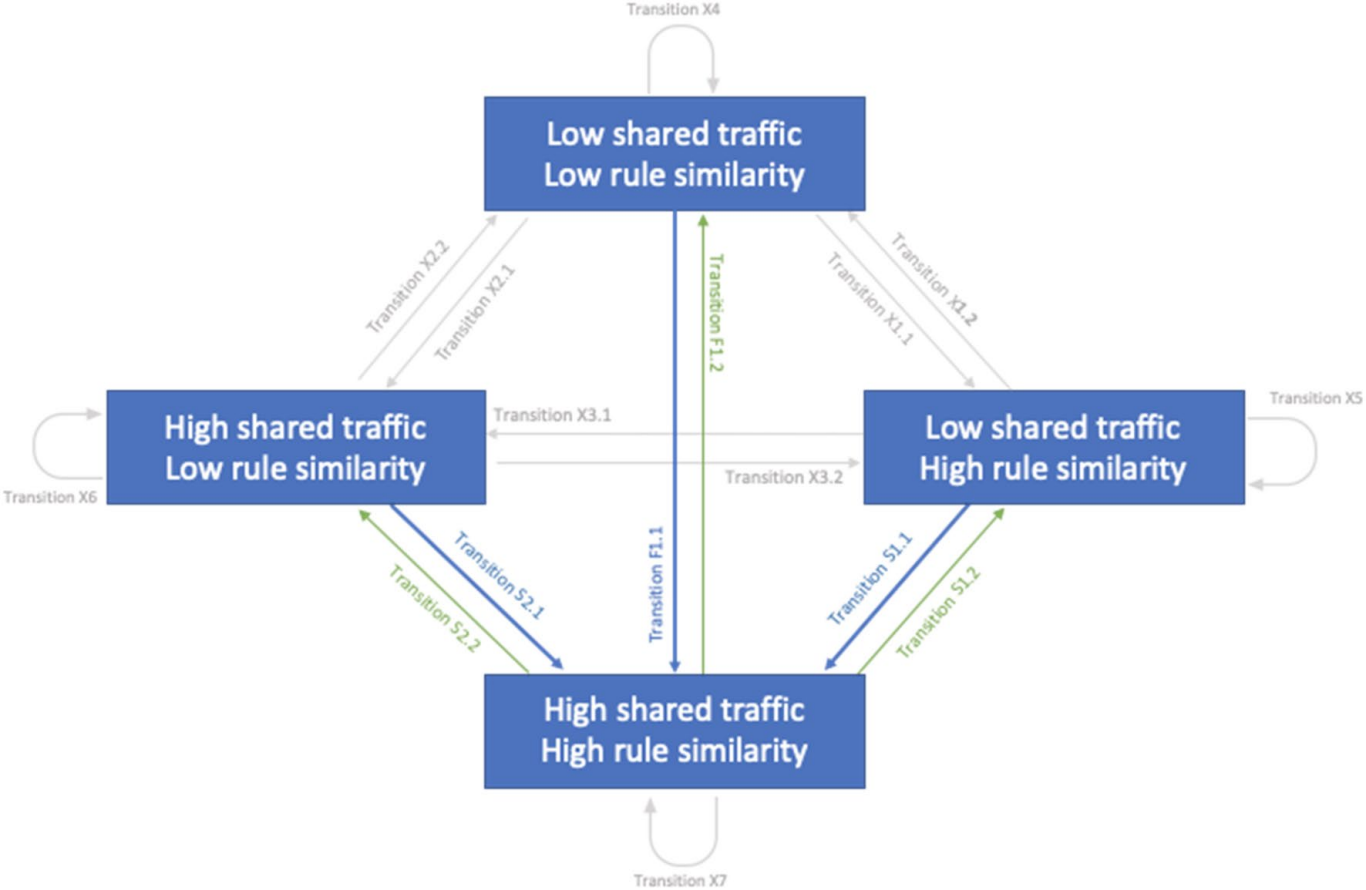
Dynamics of culture/institution interactions in terms of patterns of changes in the types of links that can connect two nodes We illustrate all possible transitions of interactions between shared traffic and rule similarities throughout the process of community development, which renders visible institutional processes over several timescales, and pits predictions of institutional effects on culture and cultural influence on institution. F represents fast-timescale transitions (in which the extent of shared governance and membership changed simultaneously in a month); S represents slow-timescale transitions (in which one changed before the other); X represents transitions that are irrelevant to the hypotheses.

We employed the dynamical multiplex spillover method that network scientists developed to quantify the co-evolution between network layers. For networks of multiple levels (in our case, similarities in different types of governance), this method provides a theoretical baseline for whether events on one network level are “spilling over” to influence another. To test our hypotheses, we identified the causal effects in multiplex networks by comparing observed patterns of multi-layer link dynamics to those predicted by the null model. Specifically links on different layers appear and disappear over time. This method uses a well-defined theoretical null rate of spillovers to detect “directional” slow-timescale spillovers (when a link change in one layer was immediately preceded by a change between the same nodes on a different layer) and “adirectional” fast-timescale spillovers (when link changes between two nodes happen on many layers simultaneously). Looking over the institutional and cultural networks simultaneously, there are 16 possible transitions (Fig. S1). Among the 16 possible transitions, we focus on a subset of greatest interest for this study. The diagram highlights some transitions that are theoretically important and others that are not, but that must be estimated as part of the model (Fig. 1).

We calculate the difference between observed and null probabilities of transitions in Fig. 1, which, on a technical level, can be interpreted as a Markov chain whose transition probabilities (arrows) between states (boxes) are both empirically and theoretically calculable. We interpret a positive (or negative) spillover as occurring between two layers when the 99% theoretical confidence interval around the difference between observed and null probabilities excludes a difference of zero. The computation details can be found in “Materials and methods”.

### Results

We begin by calculating the slow-timescale changes to investigate the central interest to this work: the spillover from culture to governance and governance to culture; the probability that two servers will gain in institutional similarity given that they share cultural similarity (Hypothesis 1, captured by S2.1 having a positive value in Fig. 1), and the probability that they will gain in cultural similarity given they already share institutional similarity (Hypothesis 2, captured by S1.1 having a positive value in Fig. 1). Then then we also investigate the fast-timescale changes to explore the co-evolution between cultural similarity and institutional similarity.

**Figure 2:**
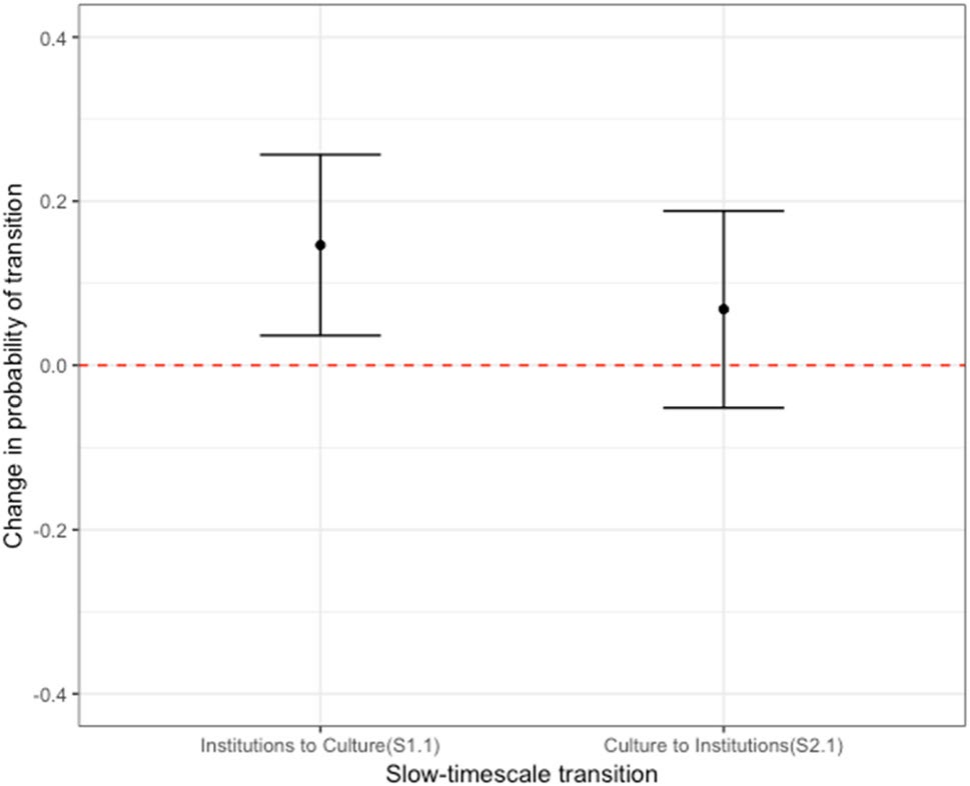
Institutional effects over cultural effects comparing to the other way round in the administrative rule type Among plugins that focus on administrative rule type, two communities with the same focus on administrative capacity are more likely to have shared traffic, while two communities with different degrees of reliance on this rule type are less likely to have a shared culture.

Here we use the example of administrative rules to illustrate the interpretation of spillover coefficients. The slow timescale processes evident in other transitions (S1.1, S1.2, S2.1 and S2.2 in Fig. 1), in which two servers were already similar on one dimension (culture or institution) and then became similar on the other, are more amenable to directional or causal interpretation. For example, S1.1 represents two servers with high rule similarity transitioning from low shared membership at one timepoint to high shared membership at the next timepoint. This transition is significantly above its expected value (*S1.1*_*diff.*_ = *0.146 [0.036, 0.256];* square brackets indicate 99% confidence interval around all statistics; See Fig. 2), whereas the spillover effects in S2.1, indicating the reciprocal effect of shared membership similarity on rule similarity, are not significant (*S2.1*_*diff.*_ = *0.068 [–0.052, 0.188])*, indicating that cultural similarities are not strong driving factors of institutional similarities. As a corroborative test of our directional hypotheses, we also check the probability that two servers similar on both dimensions will stop being similar on one dimension (S1.2 and S2.2 in Fig. 1). Our hypotheses predict a decrease: that these transitions will be observed significantly *less* often than would be expected by the null model. We find servers that are similar in one dimension are less likely to stop being similar in another dimension (*S1.2*_*diff.*_ = − *0.147 [*− *0.351, 0.056]; S2.2*_*diff.*_ = − *0.151 [*− *0.275, *− *0.028]; *Fig. S3).

**Figure 3:**
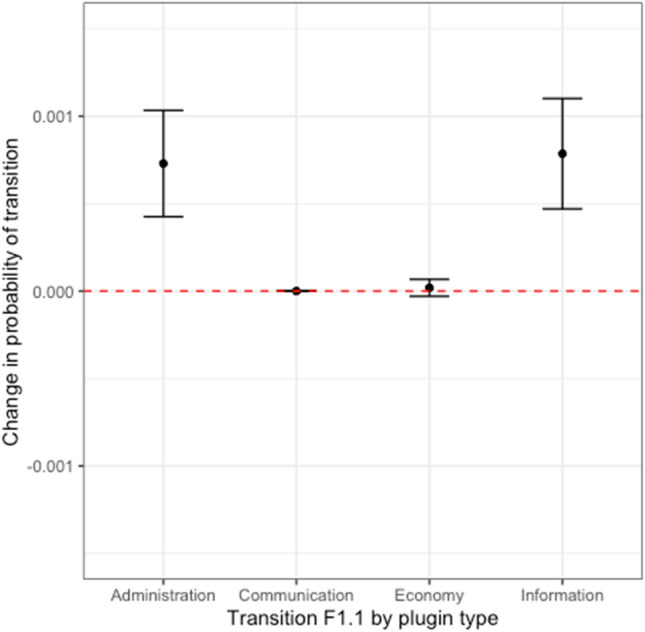
Evidence for correlations between similarities in cultural and institutional development between two communities, a common trend was for an increase from communities with low similarity to high similarity in both membership and rule type, in the administrative and informational rules among the four rule types (Transition F1.1). This figure represents those link changes from Fig. 1 that demonstrate the existence of “cross-layer” influence between our rule and cultural networks.

Looking at the fast-timescale co-transitions, institutional and cultural links appearing or disappearing simultaneously (F1.1), there is a general trend from low to high in both shared membership and rule similarity for two types of rules: the difference between the observed and null probability of transition F1.1 is greater than zero with at least 99% confidence for both administrative rules (*F1.1*_*diff.*_ = *0.0007[0.0004, 0.0010]*; see Fig. 3) and informative rules (*F1.1*_*diff.*_ = *0.0008 [0.0005, 0.0011]*). These results are ultimately consistent with a positive feedback effect. The other two rule types were insignificant: the observed/null differences were not distinguishable from zero for economy rules (*F1.1*_*diff.*_ = *0.00018 [*− *0.0003, 0.00066]*) and communication rules (*F1.1*_*diff.*_ = *0.0000003 [*− *0.0000005, 0.0000011]*). In other words, dyads that gain cultural or institutional links tend to simultaneously gain the other types of links at a higher-than-expected rate for some types of rules.

**Figure 4:**
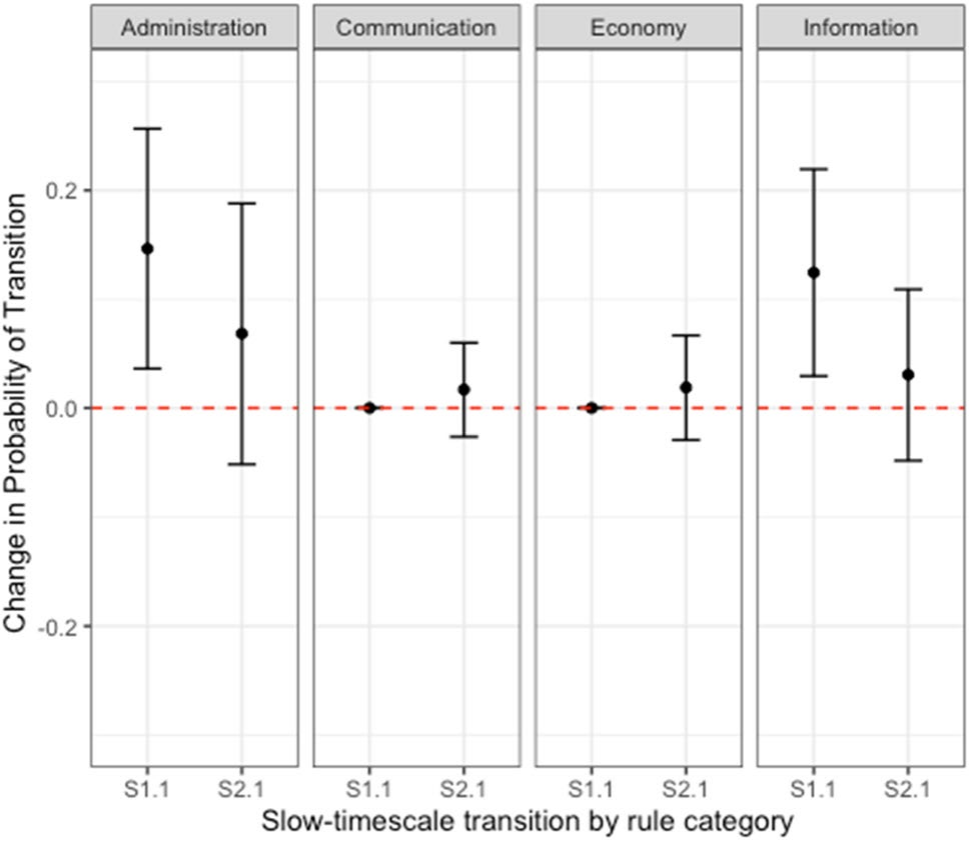
Institutional effects over cultural effects in the administrative and informational rule category. We test the effects between institution and culture for all four rule types, finding influence from institutional to cultural similarity (The relationship shown in this figure is repeated in the first panel to provide an aid to comparison). We produce the same effect for administrative and informational rules, but find no effect for communication or economic rules.

Repeating the above analysis on the other three rules types, we find the same asymmetric pattern of two servers with high rule similarity transitioning from low shared membership to high shared membership (*S1.1*_*diff.*_ = *0.124 [0.029, 0.219]*) and marginal reciprocal effects of two servers with high shared membership transitioning from low rule similarity to high rule similarity (*S2.1*_*diff.*_ = *0.0306 [*− *0.0479, 0.1091]*) for information-type rules. For communication- and economy-type rules, we find no effect in either direction (See Fig. 4).

To summarize, these results suggest that online community’s cultural and institutional dimensions are interlinked in several senses: a pair of communities that become more similar on one are significantly more likely to simultaneously become more similar on the other, and servers that are already similar in terms of governance are significantly more likely to become more similar on culture as well. All of these results hold for two rule types only: administrative and informational, with no deviations from null for any of the transitions linking economic and communication rules to the community network. Although there are many interpretations of these results, H2 is the hypothesis that is most consistent with what we observe: institutional similarities increase patterns of shared culture among *Minecraft* servers. That said, our hypotheses are not mutually exclusive, and we also find some evidence consistent with H1, namely that communities that are already similar on both governance and culture are slightly less likely than expected to become dissimilar on governance in the next time interval.

## Discussion

Our results connect and contribute to institutional theories and organizational evolution literature in three ways. First of all, it provides empirical evidence for neo-institutionalists’ view of environmental and ecological effects on institutional development^5,6,26^. The empirical findings indicate that institutional development can be due to non-functional reasons, such as shared membership with other communities. This result is then aligned with the two perspectives neo-institutionalism offers. (1) Institutional change can occur for non-functional reasons including norms and ritual. In the Minecraft example, it is the shared membership or as we theorized, shared culture. (2) Institutional development of an organization is related to the field of organizations it operates in. In this paper, we demonstrated how the institutional development of a communities can be attributed to other communities in the same exogenous environment where every community struggles for the same goals under the same pressure. The result also provides a pathway to operationalize and test DiMaggio’s theory of mimetic isomorphism. Although in this study we do not have the data or empirical evidence to reveal how institutional assimilation happens, the result of this study posits possible mechanisms of institutional isomorphism. Specifically, the effect of shared membership on institutional similarity can be understood as mutual members socially learning from each community and exchanging knowledge. Second, it offers empirical evidence for the culture-institution co-evolution literature^15,27^, demonstrating an average positive feedback effect between culture and institutions among all communities. Third, we introduce a novel method in network science to theories in organizational studies. We address the duality of organizations as cultural groups and as system of rules by analyzing organizations as units in two layers of social networks.

Although we focus on the online community context, our results, to some extent, can be generalized to real-world communities and provide some implications. The results show stronger institutional effects on culture than the other way round, even in online settings in which individuals have more bargaining power over institutional development^28^. This validates the empirical work in real-world research that supports more direct institutional effects and demonstrates that this effect is robust to changes in individuals’ agency. Another real-world implication is that administrative and informational rules can be the most effective in stabilizing and internalizing norms, even in an environment with many uncertainties and changes. Admittedly, we still need to be aware of the difference in individual interactions and the emergence of culture between online and real-world contexts when generalizing to a broader context. Specifically, cultural identity and cultural resistance in the online world might not be developed to a comparable strength within just a few months due to the nature of computer-mediated communication and the fast-evolving environment.

Although this paper provided causal pathways to the relationships between institutions and culture, it still cannot make true causal inference because it is not an experiment with random assignment. We cannot control for time-varying network-specific variables that might also influence network processes. For example, non-stationarity might affect network dynamics, which would undermine the result. Other hidden instrumental variables that correlate with changes in one layer but not with another may also alter our results.

Constraints of the method made it difficult to include in our model the effects of overall community size on shared membership. It is possible that communities with a larger size are more likely to have shared membership with others over time, which may confound the network processes.

The use of high/low splits in constructing the multiplex network can sometimes generate results that are sensitive to the choice of cutoff. It is possible that nodes near the median are more likely to be observed transition between the two categories. The median split is proven to minimize the bias generated from converting continuous variables to dichotomous measures.

We examined institutional development in *Minecraft* and its interaction with culture, which provides a framework for future institutional analysis and computational studies of cultural dynamics. The different effects of rule types provide an opportunity for future research to identify the effectiveness of specific rules. In this research, we found significant effects among administrative and informational rules, but no effects from communicative or economic rules. This result motivates valuable research questions, including how do different types of rules work? Who do they affect the most? One possible explanation is that administration and information rules promote a top-down centralized institution compared to communication and economics rules. Previous work on Minecraft communities found that only administrative and informational rules have positive effects on community survival and success in terms of attracting and maintaining members^29^, which might explain their distinctive spillover effects. Future research can investigate why players hold preferences toward different types of rules. The focus on different rules marks the institutional difference between *Minecraft* servers, which provides us several dimensions to evaluate and compare the organizational institutions.

## Conclusion

In this study, we support theories positing a positive feedback loop between culture and institution, finding, however, that institutions in *Minecraft* communities have stronger effects on culture than does culture on institutions. Specifically, we show that communities that govern themselves with similar types of rules are more likely than expected to subsequently attract similar users. Our approach to this challenge not only reveals interactions between culture and institutions but also shows the dynamic processes occurring over different timescales.

We highlight a fundamental advance that made it possible for us, in this work, to endogenize and interrelate governance and culture at the “micro” societal scale: the opportunity provided by large collections of online communities to track and compare thousands of similar but largely independent small-scale sovereign social systems.

## Methods

To quantitatively pose our questions about mutual influence, which are dynamic and involve several types of relationships between many communities, we resorted to a recent network analysis approach, the dynamical multiplex spillover method^30^. We use dynamical multiplex spillover to represent governance similarity relationships and membership similarity relationships as different networks over the same nodes (“multiplex”), and quantify the effects of link additions and deletions in one network on the corresponding links in the other (“dynamical”), relative to a theoretical null model that estimates baseline probabilities of links appearing and disappearing. Multiplex networks, also known as multilayer networks, provide a way of representing a system when nodes can be linked by links with different meanings. For example, Szell, Lambiotte, and Thurner take a multiplex approach to link entities in terms of ties denoting several different kinds of relations, including friendship, enmity, and economic partnership^31^.

The dynamical multiplex spillover is well suited to exploring the causal pathways between (1) cultural similarities between servers, measured by *shared membership*, and (2) institutional similarities between servers, measured by *shared* *server rules,* servers’ similarities in their extent of use of each institutional plugin type. Under this method, if links representing, for example, governance similarity show significant changes after a change in their membership links, that would constitute evidence in support of the hypothesis that cultural factors drive institutional factors. Of course, dynamical multiplex spillover models do not offer experiment-quality causal inference. But their use of change over time in combination with a well-formulated null model of change puts them above, for example, a design based on a simple pre/post comparison.

**Figure 5:**
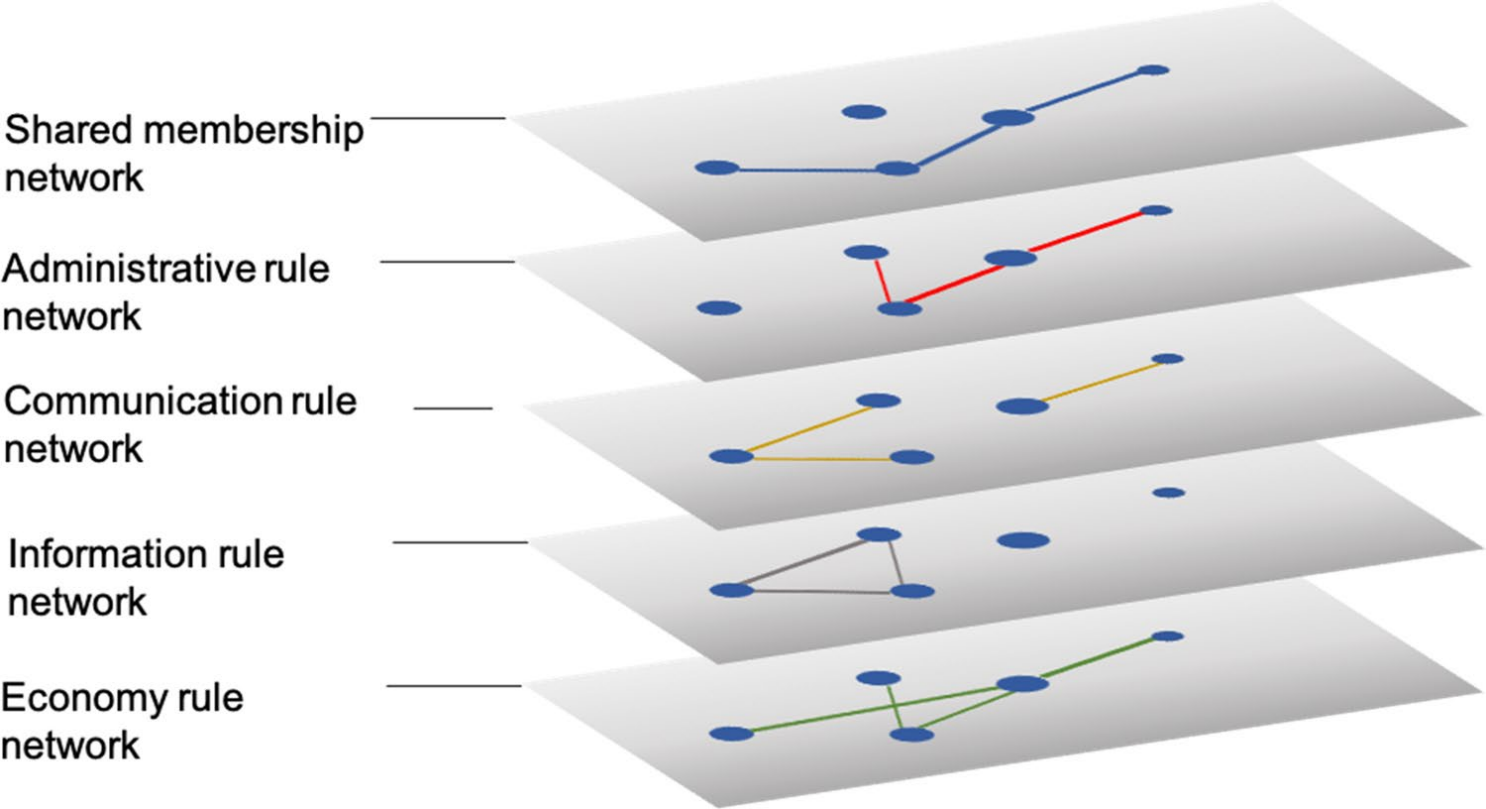
A multiplex network representation of many servers’ patterns of shared membership and common institutional structure. We layered the community network and four rule networks on top of each other to construct a multiplex network. The nodes in each layer, which signify the servers, are the same, whereas the relations in different network layers have different meanings. We consider servers to be culturally similar if they attract the same type of user. If the deletion or formation of links in, for example, the shared membership network influences the deletion or formation of links in a rule network, above baseline, then there is evidence that culture affects institutions, rather than the other way around. The position of the networks in the figure does not indicate the order of layers in the multiplex. Relational effects might occur between any of the two layers in the multiplex, but we focus on only four comparisons, those of the shared membership network with each type of rule network.

Using longitudinal data on shared membership traffic and servers’ rules, we first constructed a network of five layers (Fig. 5), combining *shared membership network* dynamics with four kinds of *server rule network*, corresponding to each of the four types of governance institution: administrator-focused mechanisms, chat mechanisms, information distribution mechanisms, and economic mechanisms. In our network, nodes signify servers, whereas links can have different meanings depending on what layer they are in. By analyzing the network processes both within and across layers in the multiplex, it becomes possible to use social network statistics to represent the idea that cultural and institutional processes interact.

We construct the shared membership traffic measure by calculating the number of users who visit two or more servers within the same month. Constrained by our statistical approach, which cannot leverage continuous link weights, we dichotomize the shared membership traffic by a median split^32^. The users with shared server membership bring to both servers their experiences and practice, which weighs in the creation and development group cultural repertoires in both servers. Therefore, servers with a high share of mutual visitors should be more similar in culture, compared to those with a low share of mutual visitors. At the same time, we use server rules to measure shared server rules. A server’s relative preferences for rules in different categories provide a proxy for its style of governance. The dynamics of rule establishment, including increasing or decreasing the number of rules of each type, proxies institutional development within each server. For each server, we create dummy variables for each of the four types of rules to characterize the servers as either high or low in each of the four types of rules. We determine “high” and “low” on the basis of a median split.

In the community network, the presence of a link indicates that two servers have a high number of shared members. In the rule networks, the presence of a link between two servers indicates that the two servers have implemented a similar number of rules of that type (i.e., similar numbers of administrative, informative, communicative, or economic plugins; see Fig. 5 below).

If culture and institution have no effect on each other, the dynamics of link appearance and disappearance in the five-layer multiplex network should be indistinguishable from the link dynamics of the five networks considered independently. On the other hand, if rules have an effect on culture, or vice-versa, we should observe that when links in the rule (or shared membership) network change, links in the shared membership (or rule) network change at a different rate than that expected from two statistically independent networks. The method draws these null predictions from the statistics of Markov chains, a well-understood theoretical model of how systems change over time.

Our approach allows quite fine-grained investigations, permitting us to ask not only what kinds of changes in one elicit changes in the other, but also the strength and direction of these interrelations, whether an increase, decrease, or persistence of links at one layer is associated with an increase, decrease, or persistence of links at another layer. This approach also required differentiating between the incremental, sequential “slow-timescale” transitions and more sudden “fast-timescale” transitions. In the type of slow time-scale transition at the root of our inquiry, a pair of nodes linked at only one layer might transition to being linked at two. By contrast, in fast-timescale transition, a pair of nodes linked at neither the membership or rule layers are in the next time-step linked on both. When a dyad changes slowly from having high similarity in one layer to having high similarity in both that and the community layer, we can interpret that within one of our directional hypotheses. But when a dyad changes from being low similarity on both layers to high on both, any direction of influence between the layers is impossible to discern. Measuring both slow-timescale and fast-timescale transition gives a full picture of the co-evolution dynamics in *Minecraft*. Of course, this same flexibility meant an explosion in the number of statistical comparisons we could perform (4 types of change in shared community link (*continued presence, continued absence, appearance, disappearance*) × 4 types of rule link change × 4 types of rule = 64 potential comparisons), a problem we use theoretical constraints and specific hypotheses to tame (Fig. 1).

## Supplementary Information

Supplementary Information.

## Data Availability

All data generated or analyzed during this study are included in Frey, S., & Sumner, R. W. (2019). Emergence of integrated institutions in a large population of self-governing communities. *PloS One*, *14*(7), e0216335^19^. (and its Supplementary Information files).
