# Supplementary material for: Institutional similarity drives cultural similarity among online communities
Source: Sci Rep. 2022 Nov 8;12:18982. doi: 10.1038/s41598-022-23223-8 (PMC9643455; doi:10.1038/s41598-022-23223-8)
Supplement: Supplementary file 1 — Supplementary Information. [file 41598_2022_23223_MOESM1_ESM.pdf]

Supplement A. Data

We analyze longitudinal user visit data from 370,000 Minecraft servers contacted through API queries bihourly between Nov 2014 and Nov 2016. The scraper accessed each server for their visitors' anonymous user ID and visit times, plugins installed, and other server features. An important feature of the Minecraft ecosystem is that user IDs persist across servers, making it possible to observe a user's trajectory across many otherwise independent communities. Following Frey and Sumner (2019), we first filtered out servers that were disconnected for the duration of data collection (~220,000), those that did not survive for at least a month (~70,000), and those that did not report full governance information (~75,000). We then further refined the resulting 5,215 servers in order to create the minimum conditions for the viability of our analysis (we address the potential effects of bias due to non-random deletion of data in the Limitations). To capture network formation and evolution continuously, we selected servers that were live for over 16 weeks in a 5-month timespan. Our analysis required us to aggregate over weeks to make the month the basic temporal unit of analysis. As the median "lifespan" of a server is 9 weeks, and administrators pay for server space monthly, the timescale of a month gives a suitable level of granularity. To match the timing of visit data on each server with the timing of rule changes, we selected servers that were operating between week 5 and week 22 in 2016 and had a final corpus of 1097 online server communities. Among these servers, we identified 2,791 unique plugins in use in the four governance plugin categories: 1,310 administration plugins, 520 communication plugins, 369 economy plugins, and 735 information plugins.

Our analysis used five months of server and user dynamics, which might seem not long enough to capture the transition and learning in *Minecraft*. Within the five months, servers might be at different stages of their life. Some servers might be at a more stable state than others, whereas some might be expanding at the time and eager to get experiences from successful neighboring servers who share members with them. Indeed, it is possible that the five months only covers certain parts of servers' life. However, the median life of *Minecraft* servers is nine weeks. Given that *Minecraft* servers evolve quickly, our assumption still holds that our data capture transitions and learning over the course of a server's life. It is also possible that the effects we observed among particular categories are due to exogenous temporal factors. Mostly, the null model we constructed already takes into account exogenous factors and produces direct correlations between different layers of networks.

Supplementary B. Figures

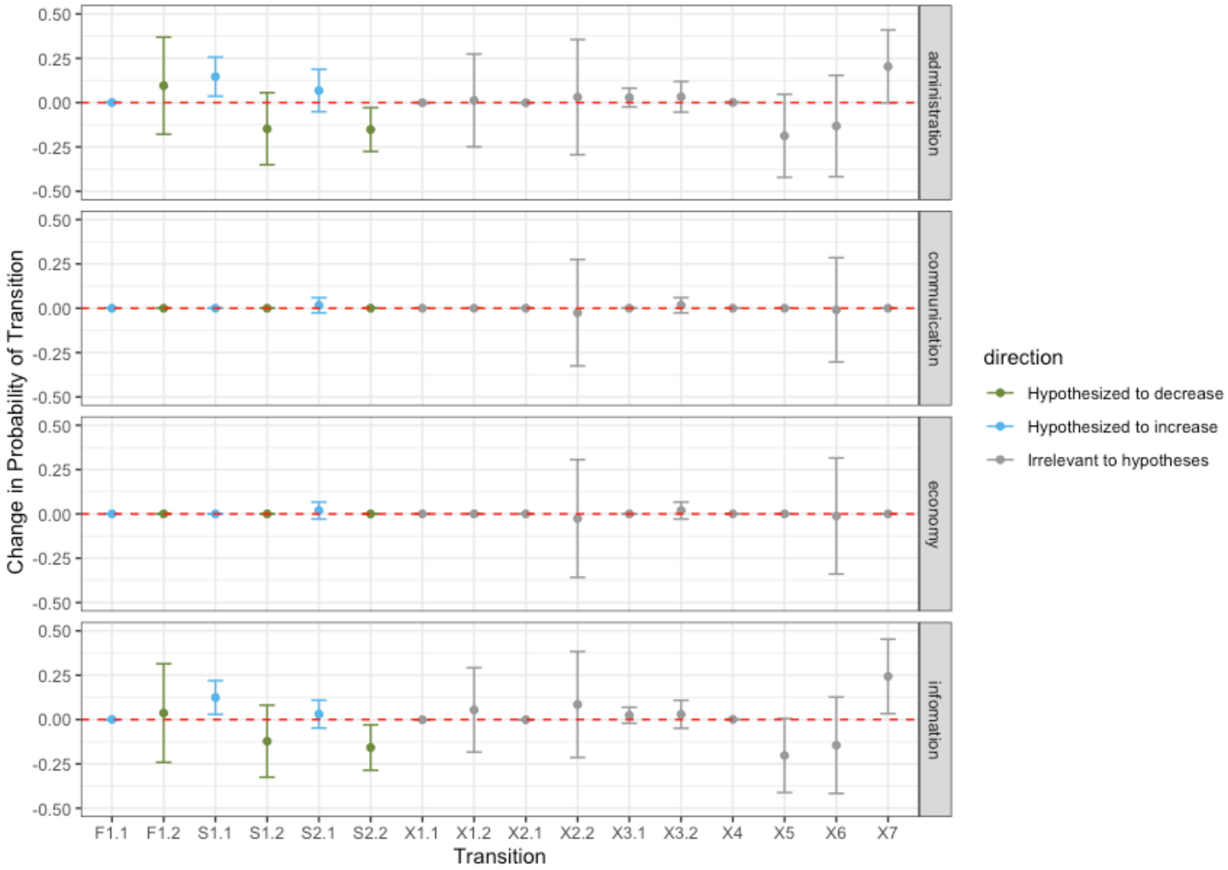

Fig. S1 All transition spillover probability within four rule categories. Across governance capabilities, we produce the effects of all transitions group by relevance to the hypotheses.

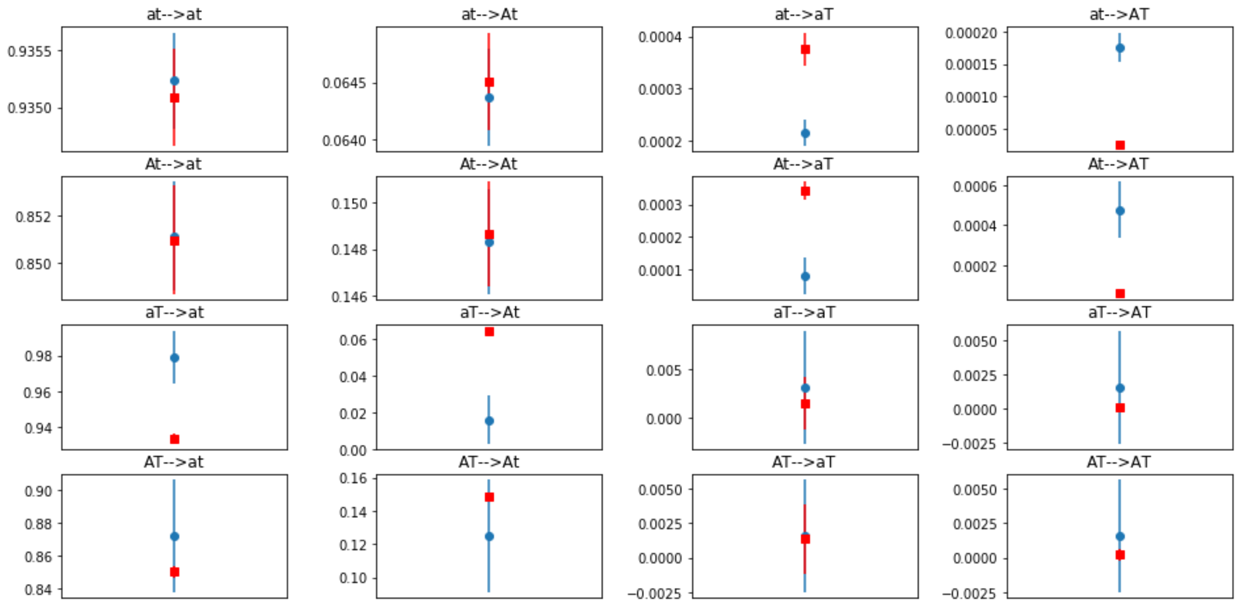

Fig. S2. Comparing mutual effects of the administrative rule network and the community network, compared to null model statistics This set of figures shows the state transition probabilities estimated for a multiplex Markov chain (blue error bar) and those of the null Markov model (red error bar), where states are the presence or absence of links across different layers of the network. This figure presents the same result as the first row of Fig. S1., the difference being that this figure shows both the null and multiplex transition probabilities, rather than their difference. We label the absence of a link with a lower-case letter. In the panel titles, lower-case a represents absent ties in administrative rule network, and lower-case t represents absent ties in community traffic network, while we indicate the presence of a link with an upper-case letter, so that A represents existing ties in administrative rule network and T existing ties in community traffic network. For example, an at → AT transition is a transition from no ties in both networks to existing ties in both. We observe a positive (negative) effect for a transition when the confidence interval around the multiplex Markov chain's estimated transition probability (blue error bar) lies entirely above (below) the confidence interval of the null (non-multiplex) Markov model's transition probability (red error bar).

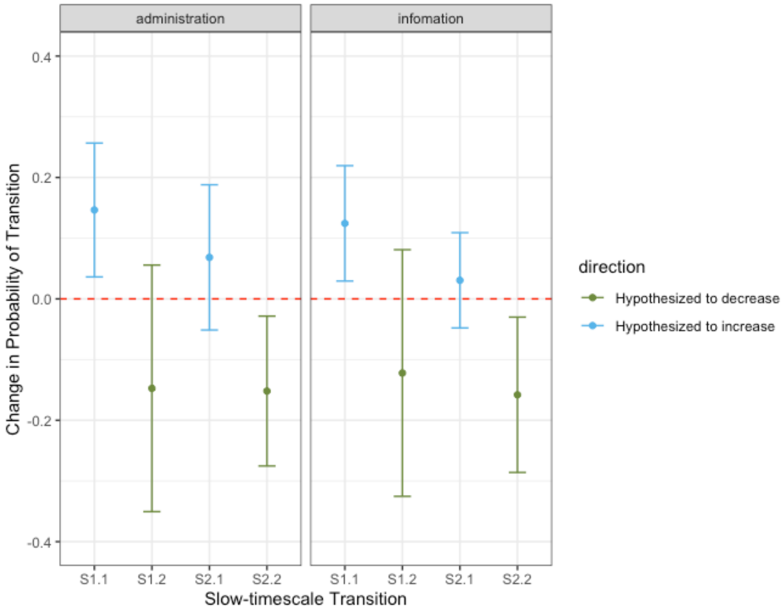

Fig. S3. For administrative and information capacities, we also produced negative effects for the opposite-direction transitions. The transition S1.2 and S 2.2 offer complementary result to S1.1 and S2.1 The positive effects in S1.1 and S2.1 indicate that servers similar in one dimension are more likely to become similar in the other dimension. Correspondingly, servers that are similar in one dimension are less likely to stop being similar in another dimension.
